# Molecular Characterization, Identification of the Volatile Organic Compounds by GC–MS, and Assessment of the Cytotoxic Activity of Leaves of *Pimenta dioica* L. Merrill Trees from Mexico

**DOI:** 10.3390/metabo15090617

**Published:** 2025-09-18

**Authors:** Isis Montalvo-López, María del Rosario García-Mateos, Juan Martínez-Solís, Ramón Marcos Soto-Hernández, Ma Carmen Ybarra-Moncada

**Affiliations:** 1 Departamento de Fitotecnia, Universidad Autónoma Chapingo, Carretera México-Texcoco km 38.5, Texcoco 56230, Estado de México, Mexico; isis.montalvolp@gmail.com (I.M.-L.); jmartinezs@chapingo.mx (J.M.-S.); 2 Área de Botánica, Colegio de Postgraduados-Campus Montecillo, Carretera México-Texcoco km 36.5, Montecillo, Texcoco 56230, Estado de México, Mexico; msoto@colpos.mx; 3 Departamento de Ingeniería Agroindustrial, Universidad Autónoma Chapingo, Carretera México-Texcoco km 38.5, Chapingo 56230, Estado de México, Mexico; mybarram@chapingo.mx

**Keywords:** allspice, cytotoxicity, dioecism, GC–MS, Soxhlet

## Abstract

**Background**: *Pimenta dioica* is a medicinal plant rich in various natural compounds, giving it significant potential for applications in the pharmaceutical, cosmetic, food, and agricultural industries. However, little is known about the metabolites present in the leaves of female and male trees, as well as their toxicity and genetic variability. Therefore, in this study, molecular characterization was conducted, the volatile compounds in the leaves of female and male trees were identified, and their cytotoxicity was assessed. **Methods**: For molecular characterization, a clustering analysis was performed using Ward’s minimum variance method; genetic distances were determined using Jaccard’s coefficient (similarity) and an analysis of molecular variance. Hexane extracts were obtained using the Soxhlet method and analyzed by gas chromatography coupled to mass spectrometry (GC–MS). The cytotoxicity of the extracts was evaluated by a bioassay with *Artemia salina*. **Results**: Forty-two metabolites were identified in leaf extracts from female and male trees, of which 17 are reported for the first time in this tissue. The female tree exhibited a distinct metabolite profile compared to the male tree and was slightly more toxic than the male tree. However, both were considered to be moderately toxic (282.00 and 222.87 μg/mL, respectively). **Conclusions**: *Pimenta dioica* has a high potential for various uses, primarily for anthropocentric purposes due to its composition of specific metabolites and moderate toxicity. The sampled trees showed a high molecular genetic variability among individuals.

## 1. Introduction

*Pimenta dioica* L. Merrill (Myrtaceae) is known as allspice, as the dried fruit flavor results from a mixture of clove, nutmeg, cinnamon, and black pepper flavors. It is an evergreen tree species, native to Mexico and Central America, growing up to 20 m tall. The leaves and fruits contain vesicles of essential oil [1]. In Mexico, the plant has been used ancestrally, primarily by the Totonaca culture, for medicinal purposes. Infusions of the leaves are used to relieve stomach pains and colic, as well as symptoms of nausea, dysentery, diarrhea, and rheumatic pain [2]. The leaf has a greater use in the preparation of various traditional Mexican dishes [3]. Mexico is the second-largest producer and exporter of *P. dioica* fruits, after Jamaica, with a planted area of 3448.70 ha and an annual production of 10,265.34 t [2,4]. The primary producing states in Mexico are Veracruz, Tabasco, Chiapas, and Puebla [5]. Despite its importance in Mexico, this crop requires further technological improvements for commercialization of the fruit, such as better harvesting techniques and appropriate soil nutrition management [5]. Because this species reaches a great height, canopy management is necessary to facilitate harvesting. The underutilized leaf tissue could increase the potential and commercial demand for this species as a source of aroma-related metabolites, instead of being used as a source of economically and medically important metabolites.

In some places, *pimienta gorda* or *xocoxóchitl* (as it is called in Mexico) is confused with black pepper (*Piper nigrum*, Piperaceae); the latter is a herbaceous climbing plant, while *P. dioica* is an evergreen tree. The fruits of both species contain some similar volatile compounds like α-thujene, α-copaene, eugenol, α-pinene, β-caryophyllene, methyl eugenol, sabinene, β-pinene, caryophyllene oxide, eucalyptol, and limonene [6]. However, the fruits from both species are morphologically different: the *P. dioica* fruits are larger, measuring up to 1 cm in diameter, while the *P. nigrum* fruits are small (up to 5 mm) and rough. As for aroma, black pepper is more pungent due to the presence of piperine, while the *P. dioica* fruits are sweeter. Although black pepper is more popular in the culinary arts, the fruits and leaves of *Pimenta dioica* also contribute aromas and flavors to foods, as well as metabolites with medicinal properties.

As its scientific name indicates, *P. dioica* has been described as a functionally dioecious hermaphrodite species [3,7], because its trees show perfect flowers; however, they are functionally different. Female trees are the only fruit producers with flowers with viable pistils and short stamens, while male trees are only pollinators, with flowers with double the amount of stamens and shorter, non-viable pistils. To identify trees, they must grow and develop for five years, and the first fruiting provides sex information [3]. Currently, the trees are differentiated, and both types are present in a 1:1 ratio; to ensure good pollination, only two male trees are left for every 10 female trees [8].

The fruit and leaves of *P. dioica* produce metabolites that make up the essential oil. In addition to the essential oil, the plant produces other metabolites such as flavonoids, phenylpropanoids, tannins, saponins, and fatty acids [9]. For this reason, it exhibits various medicinal properties, including anti-inflammatory, antidiabetic, antiseptic, antitumoral, antifungal, antioxidant, antimicrobial, anti-ulcer, and nematicidal properties [9,10]. The rich metabolite content favors the potential use of *P. dioica* in the cosmetic, food, and agricultural industries. Most studies of *P. dioica* have focused on identifying metabolites in the essential oils of its fruits and leaves. Only two reports on male and female trees provide information on volatile organic compound (VOC) composition derived by hydro-distillation and analyzed by GC–MS [11] and by SPME–GC–MS (Solid-Phase Micro-Extraction–Gas Chromatography–Mass Spectrometry) techniques [3]. In the first study conducted by Minott and Brown [11] on cultivated species in Jamaica, a similar profile of volatile components was reported in both female and male tree leaves, although their relative abundances differed. In contrast, Montalvo-López et al. [3] reported only the aroma constituents of leaves from both types of trees. Therefore, there is a growing interest in identifying additional volatile compounds that have not yet been characterized. Also, molecular studies have not been conducted to determine the genetic variability of plants for their conservation and genetic improvement. Hence, the aim of this study was to perform molecular characterization and identification of the volatile compounds of the hexane extract from leaves of female and male trees. Furthermore, the cytotoxicity of the components was tested using the BSLT (Brine Shrimp Lethality Test) method to obtain preliminary, rapid, and cost-effective information on their potential antitumor and antimicrobial activities, among others [12].

## 2. Materials and Methods

### 2.1. Plant Material Sampling

Fresh leaves were randomly sampled from the terminal branches of three female and three male *Pimenta dioica* trees from a commercial production unit located in the town of Tilzapota, Atzalan, Mexico (97° 53′10′′ W, 19° 53′10′′ N; 633 masl), in June 2023. Leaves were collected according to the four cardinal points to cover the canopy. All 18-year-old trees were subjected to uniform irrigation and nutrient management. The taxonomic identification of the species was conducted at the National Herbarium (MEXU) of the Institute of Biology at UNAM, Mexico, and the specimen was assigned the number 1531279. The tissue was dried in an oven at 45 °C until it reached a constant weight.

### 2.2. Molecular Characterization

#### 2.2.1. DNA Extraction

Fresh leaves from the three male and three female trees were disinfected with ethanol at 70% and stored at −80 °C. The CTAB-based extraction protocol, as modified by Silva et al. [13], was used for DNA extraction. CTAB 2% (Tris-HCl 10 mM at pH 8; Na_2_EDTA-2H_2_O 20 mM at pH 8.0; NaCl 1.4 M) was used as an extraction buffer. The plant tissue was macerated in liquid nitrogen; then, 1 mL of 2% CTAB was added to microtubes at 80 °C. The mixture was incubated in a thermoblock (Thomas Scientific^®^, Henry Troemmer, LLC, USA) at 85 °C for 90 min. Subsequently, the sample was centrifuged at 11,500 rpm for 10 min, and the supernatant was mixed with 500 μL of chloroform–isoamyl alcohol (24:1) in a vortex until a homogeneous mixture was obtained. Again, it was centrifuged at 11,500 rpm for 10 min. The upper aqueous phase was collected, and 900 μL of absolute ethanol, previously cooled to −20 °C, was added and mixed gently by inversion. The mixture was centrifuged at 11,500 rpm for 20 min, and the supernatant was decanted, ensuring that the pellet that formed at the bottom of the microtube was not lost. This was washed with 600 μL of ethanol (70% at −20 °C) and centrifuged for 10 min at 11,500 rpm. The pellet was allowed to dry for at least 30 min. Finally, it was resuspended in molecular-biology-grade water (50 μL). Once the DNA was obtained from the samples, the quality was evaluated by electrophoresis (integrity) and spectrophotometry (quantity and quality).

#### 2.2.2. DNA Quantification and Quality Assessment

**Figure 1:**
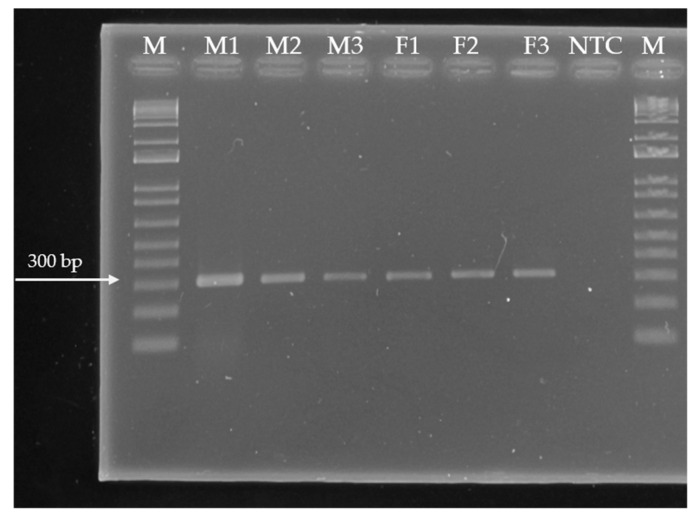
Electropherogram depicting PCR amplification products of the 16S gene (330 bp) from six DNA samples extracted from *Pimenta dioica* L. Merrill leaves. Ladder size: 100 bp. Order of paths on the gel: 1 M—size marker, 2—Male 1 accession, 3—Male 2 accession, 4—Male 3 accession, 5— Female 1 accession, 6—Female 2 accession, 7—Female 3 accession, 8—No-template control lane, 9 M—size marker.

DNA samples were quantified using a spectrophotometer NanoDrop (Thermo Fisher Scientific, DNA Factor: 50, China). For equipment calibration, 4 μL of TE buffers were used. From each sample, 4 μL of DNA dissolved in molecular-biology-grade water were taken, and absorbance was measured at a wavelength of 260 nm. Based on the concentrations obtained, each sample was diluted to a final concentration of 10 μL/mL using molecular-biology-grade water. DNA quality was evaluated through PCR amplification of a 330 bp fragment corresponding to the endogenous 16S gene (Figure 1). PCR reactions had a final volume of 25 μL. To each reaction, 2.5 μL of DNA (10 ng/μL), 1 μL of each primer (16S1: TGAGAATGGATAAGAGGCTC and 16S2: TGTTGTTCCCCTCCCAAGGG), 10 μL dNTPs, 2.5 μL PCR buffer, 1.5 μL MgCl_2_, 0.3 μL Taq DNA polymerase, and 6.2 μL of molecular-biology-grade water were added. DNA amplification was performed in a thermal cycler (Veriti Applied Biosystems^®^, Singapore) using the following conditions: initial 1 min step at 94 °C, then 35 cycles of 30 s denaturation at 94 °C, 1 min alignment at 55 °C, 1 min of extension at 72 °C, and a final extension at 72 °C for 6 s.

#### 2.2.3. ISSR PCR Reaction

Ten ISSR-type markers were selected due to their proven effectiveness in the molecular characterization of other species within the Myrtaceae family [14,15]. PCR reaction was performed to amplify loci based on the 10 ISSR markers presented in Table 1. PCR reactions for each marker had a final volume of 25 μL. To each reaction, 1 μL of DNA (10 ng/μL), 3 μL of primer, 10 μL dNTPs, 2.5 μL PCR buffer, 1.5 μL MgCl_2_, 0.3 μL Taq DNA polymerase, and 5.2 μL of molecular-biology-grade water were added. DNA amplification was performed in a thermal cycler (Veriti Applied Biosystems^®^, Singapore) using the following conditions: initial 1 min step at 93 °C, then 40 cycles of 20 s denaturation at 94 °C, 1 min alignment according to each ISSR marker (Table 1), 20 s of extension at 72 °C, and a final extension at 72 °C for 6 s.

#### 2.2.4. Electrophoresis

PCR products were separated on a 2% agarose gel in 1X TAE buffer at 100 V for 40 min. Fragment sizes were determined using the 100 bp DNA Step Ladder (DNA Ladder Thermo Fisher Scientific, Lithuania). After separation, the gel was visualized under a UV transilluminator and photographed with a gel documentation system (UVP Digidoc-It^®^ Imaging System, Upland, CA, USA). The resulting gels were analyzed by quantifying the number of polymorphic bands generated by the amplification for each primer. The presence of a band was scored as 1 and its absence as 0, thereby constructing a basic binary data matrix.

### 2.3. Extraction Procedure

The extracts were obtained with analytical-grade hexane (J.T. Baker, USA) in a Soxhlet apparatus at 70 °C for 7 h from 100 g of dried leaves from each tree to extract lipophilic components, some of which are responsible for the aroma of *P. dioica* leaves. In a round-bottom flask, 250 mL of hexane was added. The solvent was removed under vacuum using a Büchi R-210 rotary evaporator to obtain the crude extract. Each extract was flushed with nitrogen gas to preserve it and stored at −20 °C until analysis.

### 2.4. Chromatography–Mass Spectrometer Analysis

Extracts were analyzed using a gas chromatograph (Agilent Technologies 7890B, China) coupled to an autosampler (7683B) and a mass selective detector (5977A). Chromatographic separation was performed on an HP5-MS column (30 m × 0.25 mm × 0.25 µm). For each hexane extract, 5 mg was re-dissolved in 1 mL of hexane. The injection volume was 1 µL. Samples were injected in the splitless mode at 280 °C. The oven conditions were as follows: an initial temperature of 60 °C for 1 min, then increased at a rate of 6 °C/min to 270 °C, and maintained for 1 min. The column flow rate was 1 mL/min of helium. The transfer line was maintained at 300 °C throughout the analysis. The quadrupole and ionization source temperatures were 150 °C and 230 °C, respectively. Data acquisition was performed in electron impact mode over a range of *m*/*z* 50–*m*/*z* 550 in SCAN mode. The identification of compounds was carried out by analyzing the data generated by the GC–MS system (Total Ion Chromatograms), recorded using the Mass Hunter Workstation software version B.08.00 (Agilent Technologies, Inc., USA). The resulting data were processed with the Automated Mass Spectral Deconvolution and Identification System software (AMDIS v 2.71) to isolate each compound’s mass spectrum from the background. Compounds were identified by comparing their mass spectra with those in the NIST 2011 library, and some were also compared with standard compounds: p-xylene ≥ 99% (134449), α-terpineol 90% (432628), eugenol ≥ 99% (E51791), and trans-caryophyllene ≥ 98% (C9653) (Sigma-Aldrich, Saint Louis, MO, USA). The relative abundance of each compound in the extracts was obtained by integrating the peak area using the MSD ChemStation Data Analysis Application F.01.01.2317 (Agilent Technologies Inc., USA). Data presented are mean values of three repetitions for each tree.

### 2.5. Evaluation of the Extract Toxicity in Artemia salina

The bioassay was performed using *Artemia salina* (Brine Shrimp Lethality Test, BSLT), where the percentage mortality in these crustaceans was evaluated after 24 h of exposure to the plant extract to determine the LC_50_ [12]. The nauplii hatched in saline water with NaCl (35 g/L) at 25 °C after 24 h. Ten nauplii and 5 mL of saline water were placed in 25 mL vials. Hexane extracts were evaluated at three concentrations (10, 100, and 1000 ppm) and saline water was used as the control. From each extract, a stock solution was prepared by dissolving 20 mg of the extract in 2 mL of saline water supplemented with Tween 20 (0.1%); therefore, vials containing only saline water and vials containing saline water with Tween 20 (0.1%) were used as controls. Each test was performed with five replicates. The vials were kept under illumination at 25 °C for 24 h. After this time, the surviving crustaceans were counted. With the data obtained, a Probit analysis was performed to calculate the Mean Lethal Concentration (LC_50_).

### 2.6. Statistical Analysis

For the molecular statistical analysis, the Jaccard coefficient (a measure of similarity) was used to determine the genetic distances between the *P. dioica* trees. A dendrogram was obtained using Ward’s method (clustering analysis) of minimum variance. Based on Hotelling’s T_2_ pseudo-statistic [16] and pseudo-F [17], the cutoff height was determined using the SAS statistical program, ver. 9.4. To explore the hierarchical structure of genetic variation among accessions, an analysis of molecular variance (AMOVA) was applied using the Infogen^®^ package ver. 2016. A *t*-test was performed to determine whether there were significant differences in the composition of the extracts. The Mean Lethal Concentration (LC_50_) was calculated by the Probit method with RStudio ver. 2025.05.0+496.

## 3. Results

### 3.1. Molecular Characterization

**Figure 2:**
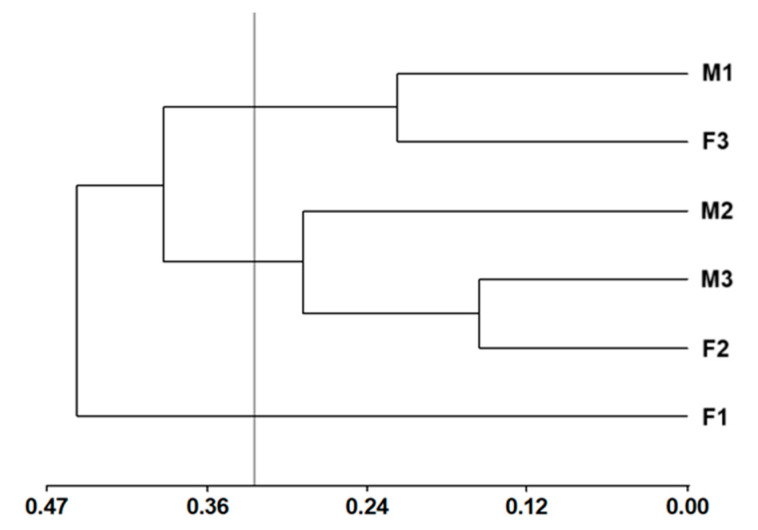
Dendrogram constructed by Ward’s minimum variance method and Jaccard’s genetic distance of three female trees (F-1, F-2, and F-3) and three male trees (M-1, M-2, and M-3) of *Pimenta dioica* L. Merrill.

The 10 primers were polymorphic (77.78%); ISSR-5 (16 bands) amplified the most bands at 61.11%, followed by ISSR-8 (12 bands) at 48.81%, ISSR-2 (10 bands) at 42.42%, and ISSR-3 (10 bands) at 60.26% amplification. Based on Hotelling’s T_2_ pseudo-statistic, the cut-off height in the dendrogram was a value of 0.53 semi-partial r^2^, which gave rise to three groups: Group I with two individuals (M-1 and F-3), Group II with three individuals (M-2, M-3 and F-2), and Group III with one individual (F-1) (Figure 2).

The primers that showed the highest polymorphic information content (PIC) values were ISSR-9 (0.36), ISSR-1 (0.34), ISSR-5 (0.33), ISSR-8 (0.32), ISSR-10 (0.32), and ISSR-2 (0.30). These primers were considered to be the most useful for further studies on the molecular characterization of allspice. The hierarchical structure of genetic variability among trees was explored using an analysis of molecular variance (AMOVA), which showed that the molecular genetic variability among accessions was very high (100%) (Table 2).

### 3.2. Yield and Chemical Profile of Leaf Extracts by GC–MS

No statistically significant differences were found in the hexane extract yield between leaves from female trees (3.13%) and male trees (4.07%). A total of 42 compounds were identified in the two extracts (Table 3). Both extracts had 29 metabolites in common. The relative abundance of each compound changed according to the type of tree; however, 32 compounds were identified in the male tree leaf extract, while a higher number (39 compounds) were identified in the female tree. These compounds were not found in the male tree extract: 4-octen-3-one; 2-methylnonane; 5-ethyl-2-methyl-heptane; 3-methyl-undecane; dodecane; α-fenchene; β-cubebene; δ-guaiene; 3,7,11,15-tetramethyl-2-hexadecen-1-ol; and 1-docosene. On the other hand, compounds not identified in the female tree extract included 5-methyl-1-undecene, (+)-epi-bicyclosesquiphellandrene, and eugenol.

**Figure 3:**
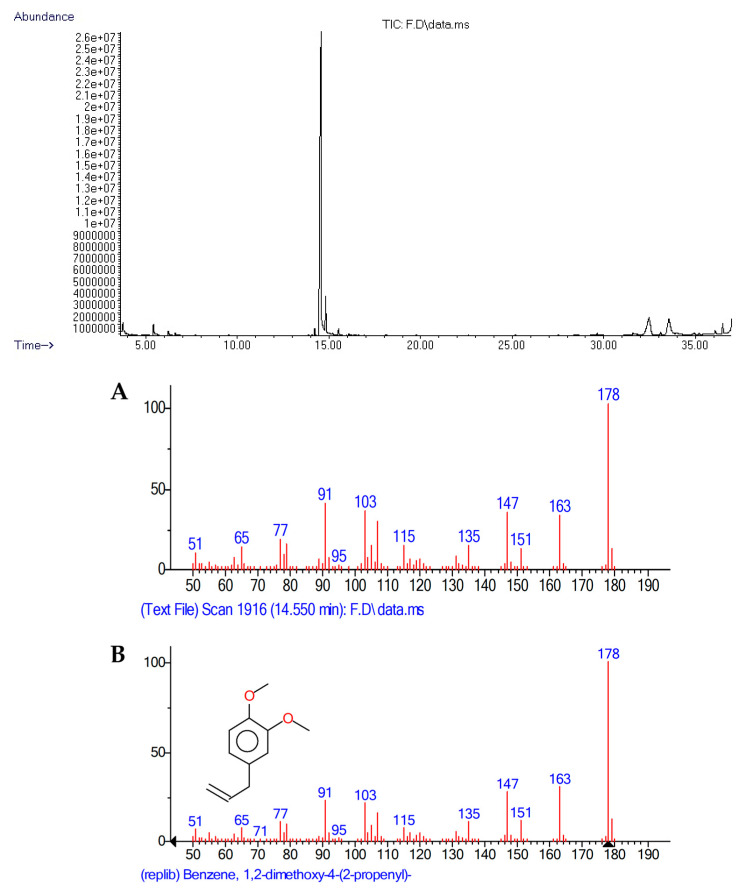
Chromatogram of the hexane leaf extract from a female *Pimenta dioica* tree and mass spectrum of the major peak: methyl eugenol. A: sample spectrum, B: NIST library spectrum.

**Figure 4:**
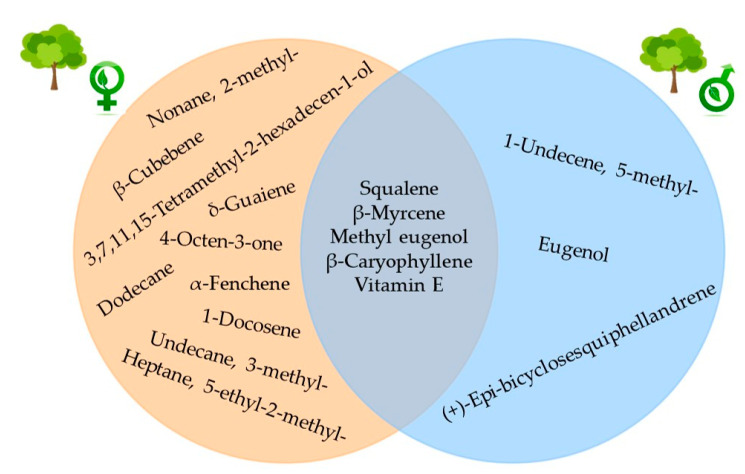
Compounds identified exclusively in the hexane leaf extract of female trees (orange) and male trees (blue) of *Pimenta dioica* L. Merrill, as well as the five major compounds present in both extracts.

According to the statistical *t*-test, β-thujene (8) was the only compound that statistically differed, with the female tree exhibiting a higher relative abundance in the leaf extract. The five main compounds in both extracts were methyl eugenol, β-caryophyllene, vitamin E, squalene, and β-myrcene (Figure 3 and Figure 4).

### 3.3. Evaluation of the Extract Toxicity in Artemia salina

The cytotoxicity values for the male and female tree leaf extracts were determined to be 222.87 μg/mL and 282 μg/mL, respectively (Table 4). According to the criteria proposed by Betancurt et al. [18], an extract with an LC_50_ greater than 1000 μg/mL is considered to be non-toxic. An LC_50_ in the range of 100–1000 μg/mL indicates moderate toxicity, while an extract with an LC_50_ of 10–100 μg/mL is classified as very toxic. Finally, an extract with an LC_50_ between 0 and 10 μg/mL is regarded as highly toxic. According to the results obtained, leaf extracts from both female and male trees were found to be moderately toxic.

## 4. Discussion

Genetic variability between female and male trees of *Pimenta dioica* has not been reported. The dendrogram revealed the formation of three groups: Group I comprised two individuals (M-1 and F-3) and Group II comprised three individuals (M-2, M-3, and F-2), with each group likely sharing the same parent. Group III contained only one individual (F-1) (Figure 1). This explains the high genetic variability detected, possibly due to the different origins of the parent trees, which provide a diverse genetic load in the descendants (individuals of the three groups). Nemara et al. [19] reported that polymorphism levels (PIC) lower than 0.25 are slightly informative, a marker with a PIC between 0.25 and 0.5 is considered moderately informative, and a PIC higher than 0.5 indicates that it is highly informative. According to Nemara et al. [19], the primers that showed the highest PIC values were ISSR-9 (0.36), ISSR-1 (0.34), ISSR-5 (0.33), ISSR-8 (0.32), ISSR-10 (0.32), and ISSR-2 (0.30). These primers could be used in further studies for the molecular characterization of this species. In the dendrogram, no grouping was observed for female and male trees, possibly due to the ISSR molecular marker used, as this marker is widely applied for genetic characterization but does not provide sufficient specificity to be employed as a sex-linked marker. The trees showed 100% variability within the samples according to the analysis of molecular variance (AMOVA); this result allowed us to infer that the six sampled trees had different progenitors, even though they were collected from the same site. Likewise, Raichand et al. [20] noted that other climatic factors, such as temperature and rainfall, may also contribute to the variability that influences plant diversity. For example, diversity is greater when rainfall is between 1000 and 1500 mm per year or greater than 1500 mm, particularly in tropical forests. In the present study, the sampling site has a mean annual rainfall of 1500 to 2500 mm and a warm humid climate, a factor that could partly explain the high variability of the analyzed trees. Raichand et al. [20] conducted a phylogenetic analysis of *P. dioica* trees from different localities in Tanzania and other species of the family Myrtaceae, explaining that the observed genetic diversity and significant genetic variations are attributed to factors such as geographic isolation, founder effects, or natural selection. Likewise, these factors could also explain the results in the present study.

Currently, no varieties of *P. dioica* have been reported in Mexico. The trees sampled in production units are wild, and few have been propagated through grafting using tree scions, which favors desirable characteristics such as large fruit, a very aromatic flavor, and disease tolerance. Therefore, these wild trees exhibit significant genetic variability, which is essential for a genetic improvement program. This allows for the selection of cultivars tailored to the needs of producers and the market, as well as the development of new cultivars with characteristics suitable for various soil and climatic conditions. Accordingly, the significant genetic variability observed explains the differences in the relative abundance and metabolite profiles in the extracts analyzed from leaves of female and male trees, as well as their relationship with the edaphoclimatic characteristics of the place of origin [20].

The studies conducted on the leaves and fruits of *P. dioica* have focused exclusively on the composition of volatile compounds and their biological activity; however, only two studies have reported the composition of volatile compounds in both female and male trees [3,11]. In contrast, other studies have documented the yield and metabolite profile of leaf extracts from the same species. According to a survey by Pino et al. [21] the yield of the hexane extract from the leaves of trees in Havana, Cuba, was reported to be 5.67%. Similarly, the yield obtained in the leaves of trees of the same species grown in India was 6.21% [22], which is higher than the yields in female trees (3.13%) and male trees (4.07%) in the present study. On the other hand, Pino et al. [21] identified seven metabolites (eugenol, thymol, carvacrol, menthol, carvone, T-cadinol, and α-cadinol) in the hexane extract profile, whereas we identified 42 compounds (Table 3). In fact, eugenol was the only common compound in both studies. These differences may be attributed to edaphoclimatic factors or genetic variability.

This study identified 42 metabolites (lipophilic) in the hexane extract in both female and male trees. In the male tree extract, 32 metabolites were found; only three compounds (5-methyl-1-undecene, (+)-epi-bicyclosesquiphellandrene, and eugenol) were not present in the female tree extract. On the other hand, 39 metabolites were identified in the female tree leaf extract, of which ten compounds (4-octen-3-one; 2-methylnonane; 5-ethyl-2-methyl-heptane; 3-methyl-undecane; dodecane; α-fenchene; β-cubebene; δ-guaiene; 3,7,11,15-tetramethyl-2-hexadecen-1-ol; and 1-docosene) were not identified in the male tree extract (Table 3). Therefore, it is important to note that plant sex is a determining factor in the synthesis of certain metabolites that partly account for their biological activity). The differences in most metabolites identified in the profiles of female and male tree leaves in studies conducted by Minott and Brown [11], Montalvo-López et al. [3], and the present study could be attributed to the extraction method employed. Minott and Brown [11] employed hydrodistillation, and Montalvo-López et al. [3] utilized the SPME–GC–MS technique; both techniques are specific to the identification of volatile compounds, unlike the Soxhlet extraction with hexane used in this study. Eugenol has been reported as the main compound in the leaves and fruits of *P. dioica* [2,11,21] and other aromatic species [23]; in the present study, it was only found in male trees, although methyl eugenol was the primary compound in both types of trees, which coincides with the results of the study by Padovan et al. [24], where they analyzed the diversity of terpenoids among species of the Myrtaceae family, showing that in the case of *P. dioica*, chemotype 1 has eugenol as the primary compound and chemotype 2 has methyl eugenol. This suggests that studies with a larger number of trees should be conducted to confirm the presence of these metabolites as chemotypes within the species, thereby contributing to chemotaxonomy and highlighting their potential for commercial and medicinal use.

Of the hydrophobic compounds in Table 3, 17 new unidentified metabolites are reported from *P. dioica* leaves (4-octen-3one; isocumene; cumene; 2-methylnonane; decane; dodecane; 5-ethyl-2-methyl-heptane; 3-methyl-undecane; α-fenchene; 5-methyl-1-undecene; vitamin E; artemisia ketone; heptacosane; δ-guaiene; 1-docosene; 1-docosene; (+)-epi-bicyclosesquiphellandrene; 3,7,11,15-tetramethyl-2-hexadecen-1-ol (phytol)). Likewise, biological activity has been reported for only a few of the 17 metabolites; however, it is essential to mention that artemisia ketone exhibits antibacterial activity [25], as well as heptacosane [26] and δ-guaiene [27]. 1-docosene possesses antioxidant and antibacterial properties [28]; (+)-epi-bicyclosesquiphellandrene possesses antimicrobial and antitumoral activities [29], and 3,7,11,15-tetramethyl-2-hexadecen-1-ol (phytol) exhibits anticancer, anti-inflammatory, antimicrobial, and antioxidant activities [30], which could partly justify the medicinal usage associated with *P. dioica*.

Most of the compounds identified in the present study have also been reported in the essential oils of leaves and fruit [9]. Due to their volatile nature, they are responsible for the characteristic odor of the species. Three major volatile compounds in both extracts in the present study had a higher relative abundance (methyl eugenol, β-caryophyllene, and β-myrcene). β-caryophyllene is a primary metabolite in *Piper nigrum* leaves, as well as linalool, β-elemene, and β-selinene; while in the fruit, α-pinene, β-pinene, limonene, myrcene, and germacrene D [31,32], all identified in the present study, have been reported. However, piperine is the metabolite responsible for the pungency of *P. nigrum.* The compounds related to its flavor and aroma include α-pinene, β-pinene, myrcene, limonene, and β-caryophyllene, among others [31], which were also identified in the present study. These profiles may explain the similarity in aromas and flavors with *P. dioica*, which is why it is commonly referred to as allspice.

Vitamin E and squalene, present in the hydrophobic extract, are part of the five primary metabolites. Vitamin E is a lipophilic compound located in the cellular lipid compartments and is therefore part of the cell membrane and lipoproteins. It stops the propagation of the chain reactions of lipid peroxidation, while, in the cell, it preserves the integrity of the membrane; therefore, it plays a crucial role in maintaining the stability of cells, such as erythrocytes and neurons in the central and peripheral nervous systems [33]. As for squalene, it is a triterpenoid with antitumoral, antibacterial, antioxidant, detoxifying, and immune-activating properties, thus demonstrating a high potential for use as an ingredient in functional foods or disease prevention [34]. Its presence partly justifies the medicinal properties attributed to the leaves of *P. dioica* since pre-Hispanic cultures.

The presence of some metabolites identified in the species justifies several of its medicinal (antifungal, antimicrobial, and nematicidal) properties [9,10]. The cytotoxicity test performed with the *Artemia salina* bioassay found that the extracts were moderately toxic (222.87 μg/mL in male tree leaves and 282 μg/mL in female tree leaves). Razak et al. [35] reported on the same species grown in India, with LC_50_ values of 102.80 and 461.52 μg/mL for the methanolic and aqueous leaf extracts, respectively. However, the differences are attributed to the polar nature of the extracts. Finally, the profile of metabolites present in plants may vary due to genetic characteristics of each species, as well as the age of the plant, the type of soil, climatic conditions, altitude, geographical location, temperature and humidity fluctuations, and rainfall are factors that could explain some chemical differences between these individuals; the sex of the tree was apparently the main factor responsible for the variation in composition [36]. Likewise, the presence of discriminant compounds between each type of tree can be considered biomarkers for their use in different industries, based on the composition and biological activity of plant extracts. Studies related to the profile of metabolites in trees of varying ages, collection times, sexes, and stages of development are required.

Among the limitations of the present study is the lack of a metabolomic analysis using different techniques (NMR and HPLC, among others) aimed at identifying other types of metabolites (flavonoids, phenolic compounds) to justify certain medicinal, antioxidant, and nutraceutical properties of interest to both producers and consumers. Furthermore, the identification of metabolites involved in signaling pathways for their synthesis would contribute to improving the yield of compounds of interest and determining the optimal harvest time for applications in the agro-food, cosmetic, and pharmaceutical industries.

## 5. Conclusions

Genetic variability was found between female and male trees. In the dendrogram, no clusters were observed that differentiate tree type. Forty-two compounds, mostly volatile metabolites and others of low polarity, were identified in the hexane extract of female trees (39 metabolites) and male trees (32 metabolites), and three compounds not present in the leaf extract of female trees were identified in the leaf extract of male trees. On the other hand, in the female tree extract profile, ten compounds were not identified in the male tree extract, demonstrating a slight difference in their biosynthetic pathways. However, 29 metabolites were present in both types of trees. Methyl eugenol was the primary compound in both extracts. Seventeen compounds were identified and are reported for the first time in *P. dioica* leaves. The diversity of compounds identified highlights the high potential of *Pimenta dioica*, with moderate toxicity observed in the bioassay using *Artemia salina*, for medicinal purposes as well as in the cosmetic and food industries.

## Figures and Tables

**Table 1:** Sequence of 10 ISSR primers and their respective annealing temperatures used for the molecular characterization of *Pimenta dioica* L. Merrill accessions.

Primer	Sequence 5′–3′	Annealing Temperature
ISSR 1	(CA)_8_AAGG	60 °C
ISSR 2	(CA)_8_AAGCT	62 °C
ISSR 3	(GA)_8_CTC	58 °C
ISSR 4	(AG)_8_CTC	58 °C
ISSR 5	(AC)_8_CTA	56 °C
ISSR 6	(AC)_8_CTG	58 °C
ISSR 7	(AG)_8_CTG	58 °C
ISSR 8	(AC)_8_CTT	56 °C
ISSR 9	(AG)_8_C	52 °C
ISSR 10	(GA)_8_ T	50 °C

**Table 2:** Molecular analysis of variance of female and male *Pimenta dioica* trees based on their ISSR molecular profiles.

Source of Variation	Degrees of Freedom	Sum of Squares	Mean Squares	*p*-Value	Variance Components	%
Between accessions	5	97.67	19.53	0.9325	1.26	100.0
Within accessions	0	0.0	0.0	<0.0001	0	0
Total	5	97.67	19.53		1.26	100.0

**Table 3:** Relative abundances (%) and metabolite profiles identified in hexane leaf extracts of male and female *Pimenta dioica* L. Merrill trees.

No.	RT	Compound	Male (%)	Female (%)
1	3.747	p-xylene ^†^	0.77 ± 0.12	1.17 ± 0.23
2	3.819	Nonane	0.37 ± 0.59	0.27 ± 0.11
3	3.953	4-Octen-3-one	nd	0.07 ± 0.01
4	4.238	Cumene	0.13 ± 0.1	0.16 ± 0.03
5	4.773	Isocumene	0.03 ± 0.00	0.04 ± 0.01
6	4.901	2-Methylnonane	nd	0.04 ± 0.01
7	4.999	Artemisia ketone	0.06 ± 0.02	0.09 ± 0.7
8	5.117	β-Thujene	0.01 ± 0.01	0.03 ± 0.02 *
9	5.420	β-Myrcene	1.89 ± 0.74	1.26 ± 0.73
10	5.586	Decane	0.38 ± 0.18	0.55 ± 0.05
11	6.044	5-Ethyl-2-methyl-heptane	nd	0.04 ± 0.03
12	6.235	Eucalyptol	1.02 ± 0.26	0.77 ± 0.23
13	6.621	β-Ocimene	0.34 ± 0.06	0.35 ± 0.17
14	7.060	3-Methyl-decane	0.03 ± 0.02	0.05 ± 0.03
15	7.497	(+)-4-Carene	0.07 ± 0.01	0.08 ± 0.02
16	7.710	Undecane	0.10 ± 0.02	0.21 ± 0.24
17	9.289	3-Methyl-undecane	nd	0.05 ± 0.04
18	9.517	1-Terpinen-4-ol	0.22 ± 0.06	0.21 ± 0.09
19	9.856	α-Terpineol ^†^	0.25 ± 0.19	0.12 ± 0.08
20	9.958	Dodecane	nd	0.15 ± 0.11
21	13.028	α-Fenchene	nd	0.02 ± 0.01
22	13.673	Eugenol ^†^	17.66 ± 35.17	nd
23	13.879	Copaene	0.68 ± 0.83	0.73 ± 0.43
24	14.220	(-)-β-Elemene	0.47 ± 0.29	0.93 ± 0.37
25	14.548	Methyl eugenol	59.20 ± 34.67	68.93 ± 2.75
26	14.821	β-Caryophyllene ^†^	6.50 ± 1.55	6.65 ± 1.11
27	15.014	β-Cubebene	nd	0.56 ± 0.23
28	15.525	α-Caryophyllene	0.84 ± 0.14	0.75 ± 0.16
29	16.088	Germacrene D	0.22 ± 0.07	0.19 ± 0.10
30	16.199	β-Selinene	0.20 ± 0.09	0.11 ± 0.04
31	16.390	α-Selinene	0.19 ± 0.08	0.13 ± 0.05
32	16.613	α-Farnesene	0.08 ± 0.04	0.11 ± 0.05
33	16.747	(+)-Epi-bicyclosesquiphellandrene	0.03 ± 0.00	nd
34	16.919	(+)-δ-Cadinene	0.16 ± 0.01	0.13 ± 0.06
35	18.129	Caryophyllene oxide	0.44 ± 0.21	0.22 ± 0.05
36	19.599	δ-Guaiene	nd	0.25 ± 0.12
37	20.905	5-Methyl-1-undecene	0.05 ± 0.01	nd
38	22.762	3,7,11,15-Tetramethyl-2-hexadecen-1-ol	nd	0.12 ± 0.03
39	33.528	Vitamin E	3.53 ± 1.47	9.18 ± 3.23
40	34.942	Heptacosane	0.77 ± 0.54	0.69 ± 0.71
41	36.079	1-Docosene	nd	1.25 ± 1.25
42	36.496	Squalene	3.32 ± 0.60	3.31 ± 3.40

^†^ Compared to a standard reference; nd: not detected. Values were compared using *t*-test. An asterisk (*) indicates a statistically significant difference (*p* < 0.05). Comparisons were conducted within rows. Data are presented as mean ± standard deviation.

**Table 4:** Significant parameters of the probit model for the determination of LC_50_ of the hexane leaf extracts from female and male *Pimenta dioica* trees.

Leaf	b	*p*-Value	e	*p*-Value
Male	−1.2653	1.355 × 10^−10^	222.872	6.550 × 10^−12^
Female	−1.5747	2.536 × 10^−6^	282.000	1.191 × 10^−6^

## Data Availability

The original contributions presented in this study are included in the article. Further inquiries can be directed to the corresponding author.
